# Co-complexes on modified graphite surface for steady green hydrogen production from water at neutral pH

**DOI:** 10.3389/fchem.2024.1469804

**Published:** 2024-09-25

**Authors:** Esteban A. Toledo-Carrillo, Mario García-Rodríguez, Emilia Morallón, Diego Cazorla-Amorós, Fei Ye, Varun Kundi, Priyank V. Kumar, Oscar Verho, Joydeep Dutta, Bjorn Åkermark, Biswanath Das

**Affiliations:** ^1^ Department of Applied Physics, KTH Royal Institute of Technology, Stockholm, Sweden; ^2^ Departamento de Química Física e Instituto Universitario de Materiales, Universidad de Alicante, Alicante, Spain; ^3^ Departamento de Química Inorgánica e Instituto Universitario de Materiales, Universidad de Alicante, Alicante, Spain; ^4^ School of Chemical Engineering, University of New South Wales, Sydney, NSW, Australia; ^5^ Department of Medicinal Chemistry, Biomediciniskt Centrum BMC, Uppsala University, Uppsala, Sweden; ^6^ Department of Organic Chemistry, Arrhenius Laboratory Stockholm University, Stockholm, Sweden

**Keywords:** green hydrogen, water reduction, molecular electrodes, cobalt, sustainable energy, electrocatalysis

## Abstract

Green hydrogen production from water is one attractive route to non-fossil fuel and a potential source of clean energy. Hydrogen is not only a zero-carbon energy source but can also be utilized as an efficient storage of electrical energy generated through various other sources, such as wind and solar. Cost-effective and environmentally benign direct hydrogen production through neutral water (∼pH 7) reduction is particularly challenging due to the low concentration of protons. There is currently a major need for easy-to-prepare, robust, as well as active electrode materials. Herein we report three new molecular electrodes that were prepared by anchoring commercially available, and environmentally benign cobalt-containing electrocatalysts with three different ligand frameworks (porphyrin, phthalocyanine, and corrin) on a structurally modified graphite foil surface. Under the studied reaction conditions (over 7 h at 22°C), the electrode with Co-porphyrin is the most efficient for the water reduction with starting ∼740 mV onset potential (OP) (vs. RHE, current density 2.5 mA/cm^2^) and a Tafel slope (TS) of 103 mV/dec. It is followed by the molecular electrodes having Co-phthalocyanine [825 mV (OP), 138 mV/dec (TS)] and Vitamin-B_12_ (Co-corrin moiety) [830 mV (OP), 194 mv/dec (TS)]. A clear time-dependent improvement (>200 mV over 3 h) in the H_2_ production overpotential with the Co-porphyrin-containing cathode was observed. This is attributed to the activation due to water coordination to the Co-center. A long-term chronopotentiometric stability test shows a steady production of hydrogen from all three cathode surfaces throughout seven hours, confirmed using an H_2_ needle sensor. At a current density of 10 mA/cm^2^, the Co-porphyrin-containing electrode showed a TOF value of 0.45 s^−1^ at 870 mV vs. RHE, whereas the Co-phthalocyanine and Vitamin-B_12_-containing electrodes showed 0.37 and 0.4 s^−1^ at 1.22 V and 1.15 V (vs. RHE), respectively.

## 1 Introduction

Efficient electrochemical production of hydrogen (H_2_) from water under mild conditions at a close to neutral pH is vital from the perspective of sustainable green energy production (Kandemir et al., 2016; Xie et al., 2019; Hoque et al., 2020; Shiva Kumar and Lim, 2022; Das et al., 2023; Zainal et al., 2024).

The state-of-the-art catalysts for the Hydrogen Evolution Reaction (HER) are Pt-based, which increases the overall cost of the system due to the scarcity of noble metals like Pt (Faber and Jin, 2014; Toledo-Carrillo et al., 2024). Thus, many studies have been conducted for searching of novel low-cost electrocatalysts, based on a variety of non-precious earth abundant transition metals, including Fe, Co, Ni, Cu, Mn, and their complexes (Sun et al., 2020; Wang et al., 2020; Makhado et al., 2021). Manganese-, iron- and cobalt-based transition metal oxides have been found to have high electrocatalytic activity because of the existence of various oxidations states, high conductivity, and excellent structural durability (Moni et al., 2017; Sun et al., 2017; Zhu et al., 2018). Nickel transition metal has also been reported to be active for HER; Li M. et al. (2019) applied ZnO nanorods supported nickel foam (NF) as the template to fabricate Ni_3_ZnC_0.7_/NCNT (NCNT: nitrogen-doped carbon nanotube) arrays for water splitting; and Elakkiya et al. (2019) fabricated a flower-like nanoporous-NiCo_2_O_4_ material as electrocatalyst for the water splitting in alkaline media, which displayed a high mass activity with low overpotentials, and small Tafel slopes due to its unique surface and electronic band structure with abundant active sites and porosity, electrochemical active surface area, rapid electron transfer and structural durability. Other elements such as Mo (Li R. et al., 2019), Ni-Co (Zhang et al., 2019), Ni-Co-Mo (Hu et al., 2019) and Mo-Mn (Gong et al., 2019) alloys, and Ir-Ag (Zhu et al., 2019) nanotubes show also promising results. Furthermore, to expose more active sites and increase the electric conductivity, it is an effective way to coat the electrocatalysts on the three-dimensional (3D) conductive support. For instance, Xu et al. (2019) successfully anchored the integrated hybrid electrocatalyst of NiFe_2_O_4_ nanoparticles directly on the vertically aligned carbon nanotubes showing enhanced electrocatalytic performance. This is attributed to the synergistic effect raising from the interaction of metal nanoparticles and the carbon nanotubes.

The importance of the conductive support is highlighted in a number of reports utilizing cobalt-containing molecular electrocatalysts. (McAlpin et al., 2010; Kim et al., 2015; Kandemir et al., 2016; Yang et al., 2017; Das et al., 2022). For instance, vitamin B_12_ (VB_12_), a cobalt-containing naturally abundant catalyst, has been successfully tested for several electrocatalytic reactions, including electrochemical processes such as water oxidation, proton reduction, and carbon dioxide reduction (Giedyk et al., 2015; Jia et al., 2020). Although the redox-active corrin-ligand framework in VB_12_ allows the cobalt center to readily shuttle between +3 and +1 oxidation states, it remains challenging to modify the substituents on the ring. Small molecules with simpler ligand frameworks as alternatives allow easier structural modification for stepwise improvement, e.g., metal complexes with porphyrins and phthalocyanines (Kärkäs et al., 2014; Das et al., 2019). These complexes with various electron-donating or withdrawing groups in the periphery have been studied due to their easy handling and electrocatalytic response.

**FIGURE 1:**
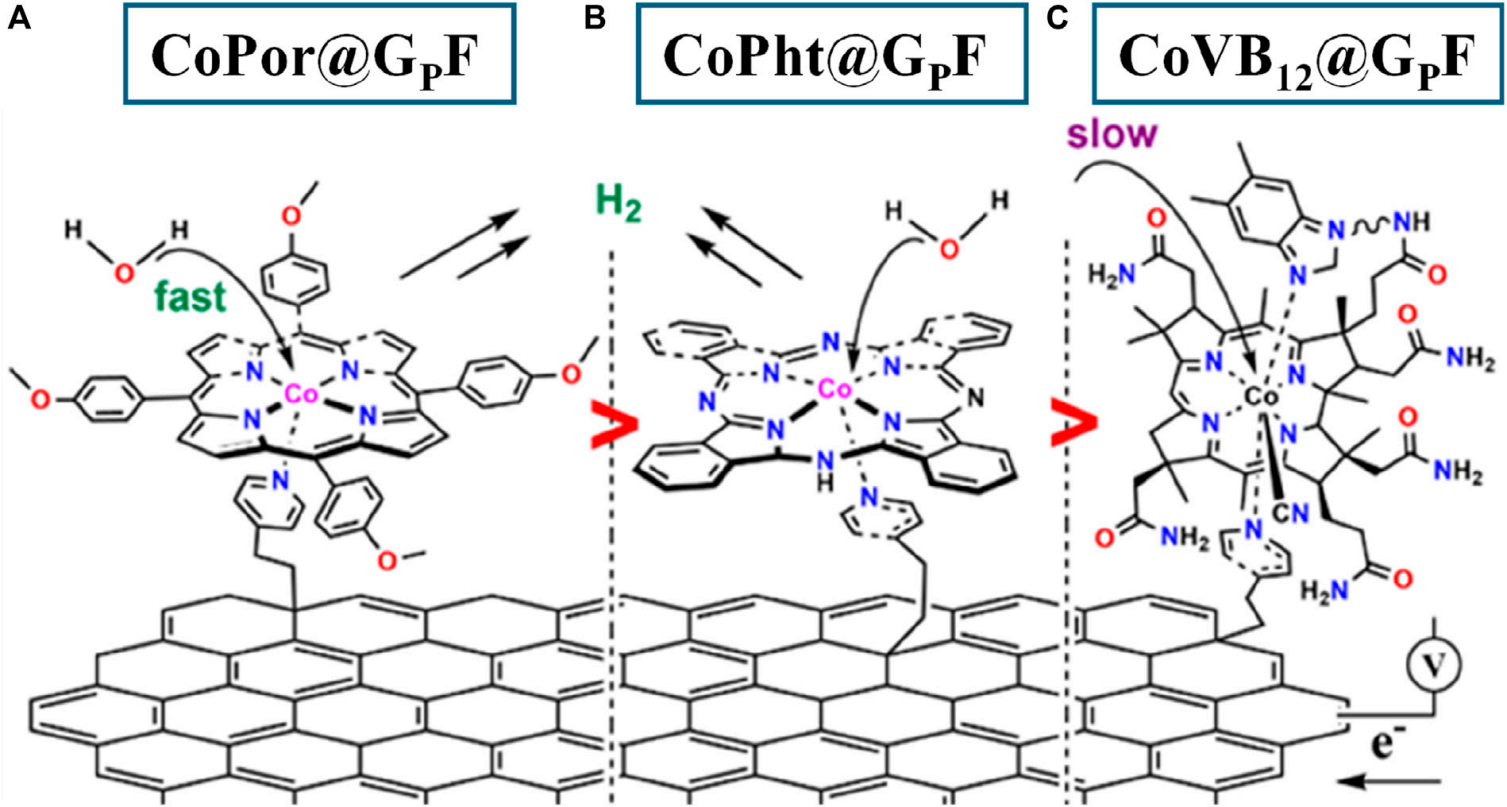
Pictorial representation of the electrodes [CoPor@G_P_F **(A)**, CoPht@G_P_F **(B)**, and CoVB_12_@G_P_F **(C)**] that are studied in this project.

Molecular electrocatalysis allows detailed structural understanding, modification, and optimization which is hard to obtain from metal oxides-assisted electrocatalytic processes due to the comparatively lesser understanding of the structure and morphology of the latter (Copéret et al., 2003). However, molecular systems undergo deactivation due to decomposition and polymerization in solution. Metal-leaching and/or copolymerization are other common disadvantages of molecular inorganic catalysts. This can be avoided by anchoring them on the modified solid conductive surface through a suitable linker (Hoque et al., 2020; Gil-Sepulcre et al., 2021; Ventosa et al., 2021; Das et al., 2023). These molecular electrodes allow structural understanding at the molecular level as well as provide higher stability of the catalysts. Herein we present three cobalt-containing electrocatalysts with corrin (vitamin B_12_), porphyrin, and phthalocyanine ligand frameworks (Figure 1) which are anchored on pyridine-modified graphite foil (G_P_F), and their electrocatalytic hydrogen production performance at the neutral pH (pH 7.1, 0.1 M phosphate buffer) conditions was investigated. From the cumulative electrocatalytic results, it is clear that the Co-porphyrin system on the modified graphite surface is by far the best electrode for hydrogen production (with −743 mV vs RHE) through direct water reduction in terms of onset potential (−740 mV overpotential at 2.5 mA/cm^2^) but also in terms of stability, showing a stable potential for up to seven hours at 10 mA/cm^2^.

The results are supported by X-ray photoelectron spectroscopy (XPS), cyclic voltammetry (CV), linear sweep voltammetry (LSV), electrochemical impedance spectroscopy (EIS), UV-Vis spectroscopy, transmission electron microscopy (TEM) and scanning electron microscopy (SEM).

## 2 Results and discussion

### 2.1 Synthesis

To covalently link the electrocatalysts on conductive graphite support, surface modification was done utilizing a diazotization reaction involving 4-(2-aminoethyl) pyridine, isoamyl nitrite, tetrafluoro boric acid, and acetic acid (see Supplementary Material for detailed procedure) (Das et al., 2022; Das et al., 2023). This resulted in a pyridine-modified graphite foil (G_P_F) that allows free pyridyl groups to form covalent bonding interactions with the molecular electrocatalysts while being linked to the graphite surface through a -CH_2_-CH_2_- unit. This technique of structural modification of the carbon cloth is tested and optimized for large-scale production which results in a minimal amount of waste (Das et al., 2023).

Three separate 10 mL 0.5 mM solutions (Water: Ethanol 1:4) of cobalt (II) tetrakis (4-methoxyphenyl) -porphyrin, cobalt (II) phthalocyanine, and vitamin B_12_ were prepared and degassed with nitrogen for 5 min before using for catalyst anchoring. To covalently attach the electrocatalysts, 2 cm × 2 cm G_P_F was immersed into the freshly prepared solutions, and the container was kept at 65°C for a period of 6 h under continuous slow stirring and a nitrogen atmosphere. It was followed by careful collection of the electrodes and washing them with cold water (3 × 1 mL) and acetone (2 × 1 mL) before drying them in a 60°C oven overnight. It resulted in the molecular cathodes CoPor@G_P_F (Figure 1A), CoPht@G_P_F (Figure 1B), and CoVB_12_@G_P_F (Figure 1C).

### 2.2 Characterization

**FIGURE 2:**
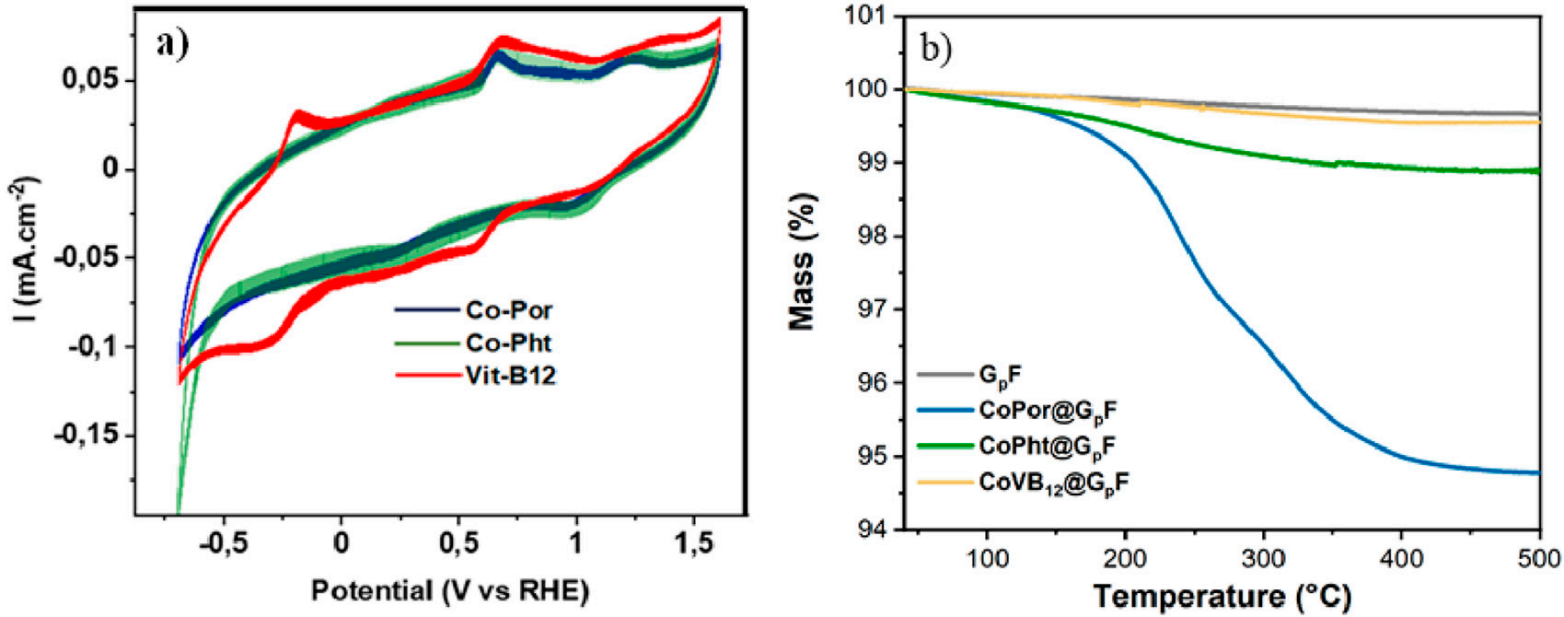
**(A)** Cyclic voltammogram of 1 mM cobalt (II) tetrakis (4-methoxyphenyl) -porphyrin (Co-Por), cobalt (II) phthalocyanine (Co-Pht), and vitamin B_12_ (Co-VB_12_) in pH 7.1 phosphate buffer (0.1 M) solution. 5 v% of ethanol was added in the case of Co-Por to completely solubilize this sample. **(B)** Thermogravimetric analysis (TGA) of G_p_F and all three electrodes CoPor@G_P_F, CoPht@G_P_F, and CoVB_12_@G_P_F.

All three molecular catalysts are commercially available with >99% purity and no further purification was performed before anchoring them onto G_P_F. The electrochemical responses of CoPor, CoPht, and CoVB_12_ were investigated in pH 7.1 phosphate buffer (Figure 2A) prior to their anchoring to graphite foil. Both Co-Por and Co-Pht showed two quasi-reversible redox waves within the potential range of 0.70 V and 1.25 V whereas Co-VB_12_ showed two well-resolved reversible oxidation waves at around −0.25 and 0.6 V. Co-VB_12_ also showed another oxidation wave at around 1.25 V. Interestingly, Co-Pht showed a sharp water reduction feature at around −0.6 V, which was absent in case of Co-Por and CoVB_12_. The redox peaks can be associated with the redox behavior of the cobalt species and also with oxidation/reduction reactions in the π-system ring to form the corresponding radical (Zagal et al., 1992; Sun et al., 2003; Calabuig-Mompó et al., 2024). Previously reported systems have identified that the molecular catalysts can undergo decomposition upon prolonged electrolysis and might not be reliable in case of prolonged electrocatalysis (Lee et al., 2019). The current study investigates the reactivity of these molecular catalysts after they are anchored to modified graphite foil using covalent bonding through pyridyl units. The electrocatalysis was tested for continuous hydrogen evolution (via water reduction) over a period of seven hours.

The loading of the catalyst on the graphite foil was studied by thermogravimetric analysis (TGA). Figure 2B shows the weight changes in the range between 30°C–500°C. With similar systems a clear weight loss was observed between 200°C–300°C associated to the desorption of the electrocatalyst, as reported previously (Das et al., 2022). The partial weight reduction as compared to the substrate material reveals a loading of 4.9%, 0.9% and 0.2% for CoPor@G_P_F, CoPht@G_P_F, and CoVB_12_@G_P_F, respectively in the current study. This technique reflects a stronger anchor between the G_P_F and the active phase of the electrocatalyst in the CoPht@G_P_F and CoVB_12_@G_P_F samples compared to the CoPor@G_P_F sample, which presumably has an influence on the electrocatalytic activity (Zhang and Wang, 2021).

The structural identity of CoPor@G_P_F, CoPht@G_P_F, and CoVB_12_@G_P_F were investigated by XPS, SEM, and TEM spectroscopy. XPS analysis was performed before and after exposing the samples to a 0.1 M phosphate buffer electrolyte overnight (Supplementary Figure S1), since it is expected that the species composing the electrocatalyst change upon contact with the electrolyte. The N 1s and O 1s spectra revealed significant differences, but no changes were observed in the C 1s spectrum. The Co 2p spectrum was only defined in the CoPht@G_P_F sample, but Co 2p presence in the CoPor@G_P_F and CoVB_12_@G_P_F samples could be confirmed. The immersion into the electrolyte solution (pH 7.1 phosphate buffer) did not alter the Co 2p spectrum. The main differences resulting from the impregnation with the electrolyte were a 4%–5% increase in O in all samples and a 1% increase in N. Supplementary Tables S1 and S2 show the mass and atomic percentage of all samples.

**FIGURE 3:**
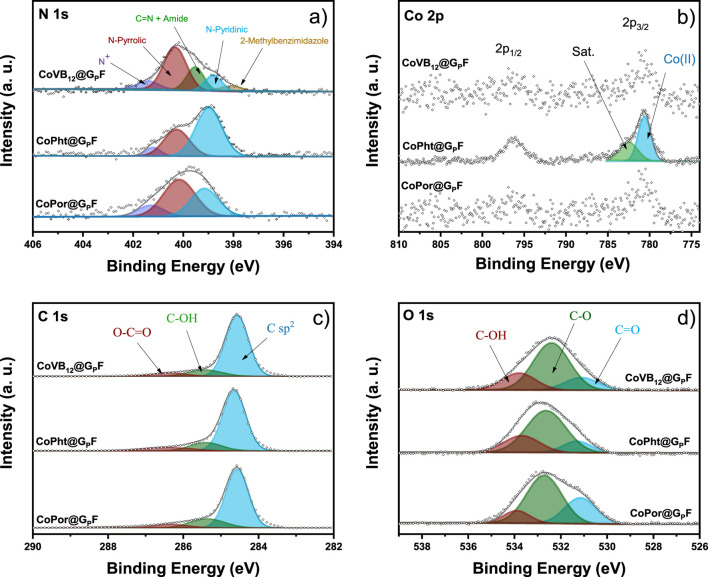
XPS analysis [**(A)** N 1S analysis, **(B)** Co 2P analysis, **(C)** C 1S analysis and **(D)** O 1S analysis] of the three electrodes CoPor@G_P_F (bottom), CoPht@G_P_F (middle), and CoVB_12_@G_P_F (top) after the impregnation with 0.1 M phosphate buffer (pH 7.1).

To analyse the N and O species in the compounds, the spectra were deconvoluted (Supplementary Table S1 shows the deconvolution parameters applied). Figure 3 displays the deconvolution of the spectra from the impregnated samples, providing a more accurate representation of the catalyst’s composition during the reaction. In contrast, the deconvolution of the spectra without immersion can be observed in Supplementary Figure S2. The percentages of the deconvolution of each species can be seen in Supplementary Table S2. A higher concentration of cobalt species from the ligand framework was detected in the CoPht@G_P_F sample. Considering that the same treatment was used to anchor the ligand frameworks to the graphite, it is possible to affirm that there is a higher affinity of the phthalocyanine molecules to be anchored to the graphite compared to the porphyrin and VB_12_ molecules. The high-resolution N 1s spectrum of CoPor@G_P_F (Figure 3A) reveals three signals. The signal at ∼398.9 eV corresponds to the N-pyridine linker to graphite. The signal at ∼400.1 eV represents three overlapping possibilities: N-Co bond due to Co addition, N-pyrrole, and N-imine bond resulting from inadequate Co incorporation (Macquet et al., 1978; Mette et al., 2016). The signal at ∼401.3 eV is associated with positively charged nitrogen atoms, a common outcome of X-ray radiation during measurement (Alemany-Molina et al., 2022). CoPht@G_P_F also has three contributions similar to the CoPor@G_P_F sample, with the addition of a new signal from the N-pyridinic-like bond from phthalocyanine (Artyushkova et al., 2008; De Riccardis et al., 2020). CoVB_12_@G_P_F shows 5 peaks at ∼398.1 eV (2-methylbenzimidazole), ∼398.8 eV (N-pyridyl), ∼399.5 eV (CN and amide bond), ∼400.3 eV (N-Co and N-pyrrolic), and ∼401.4 eV (N^+^) (Saravanan et al., 2020; Jia et al., 2020).

The high-resolution O 1s spectrum of the three sample types (Figure 3) was deconvoluted and shows three contributions at ∼531.2 eV, ∼532.6 eV, and ∼533.9 eV, associated with C=O, C-O and C-OH, respectively (Peebles et al., 2017; Saravanan et al., 2020). The electrolyte causes differences with a rise in the C=O ratio (Supplementary Table S3), particularly in the CoPor@G_P_F sample that increases by almost 20%. Subsequently, the deconvolution of the Co 2p spectrum for the CoPht@G_P_F sample reveals a peak at 780.8 eV, which is associated with the presence of a multilayer of phthalocyanine with adsorbed Co^2+^ on a graphite surface. Additionally, a satellite peak is detected at 782.6 eV, which is attributed to the presence of a sub-monolayer of adsorbed phthalocyanines. This peak has been observed for various combinations of adsorbates and substrates (Gottfried and Marbach, 2009; Petraki et al., 2010; Schmid et al., 2012).

When comparing the samples, a higher contribution of N-pyrrolic groups is observed in the CoPor@G_P_F and CoVB_12_@G_P_F samples (accounting for nearly 50%), while in the CoPht@G_P_F sample, the predominant group is associated with N-pyridinic groups (approximately 61%). Regarding surface oxygenated groups, all samples show a substantial presence of C-O groups, nearly 60%. However, it is noteworthy that the CoPor@G_P_F sample exhibits a higher ratio of C=O species, approaching 30% (Supplementary Table S3).

**FIGURE 4:**
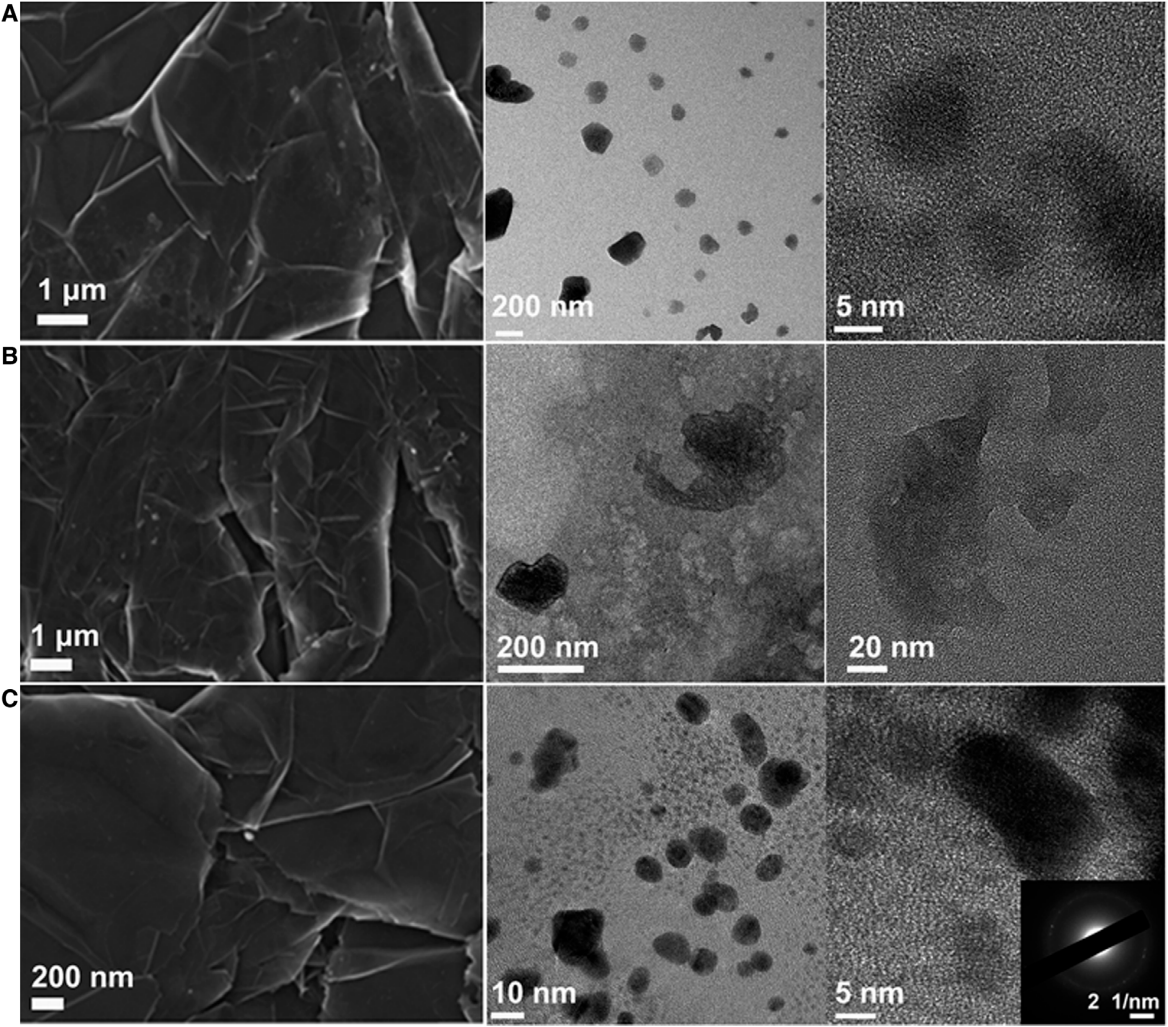
SEM images of the active surfaces of the three electrodes: CoVB_12_@G_P_F **(A)**, CoPht@G_P_F **(B)**, and CoPor@G_P_F **(C)**.

The electrodes were further studied by scanning electron microscopy (SEM), as depicted in Figure 4. The surface of the pristine graphite substrate presents a wrinkled surface with the clear appearance of graphene layers. The molecular electrode surfaces (after anchoring the electrocatalysts on G_P_F), show a similarly smooth surface as the pristine graphite, but with the presence of small aggregates with sizes lower than 100 nm, evidencing the anchoring of the Co-complexes in all three cases, with a well-conserved graphite surface during the synthesis process, which is of great importance to ensure the surface electrical conductivity is conserved as no clear evidence of surface oxidation/exfoliation is observed.

**FIGURE 5:**
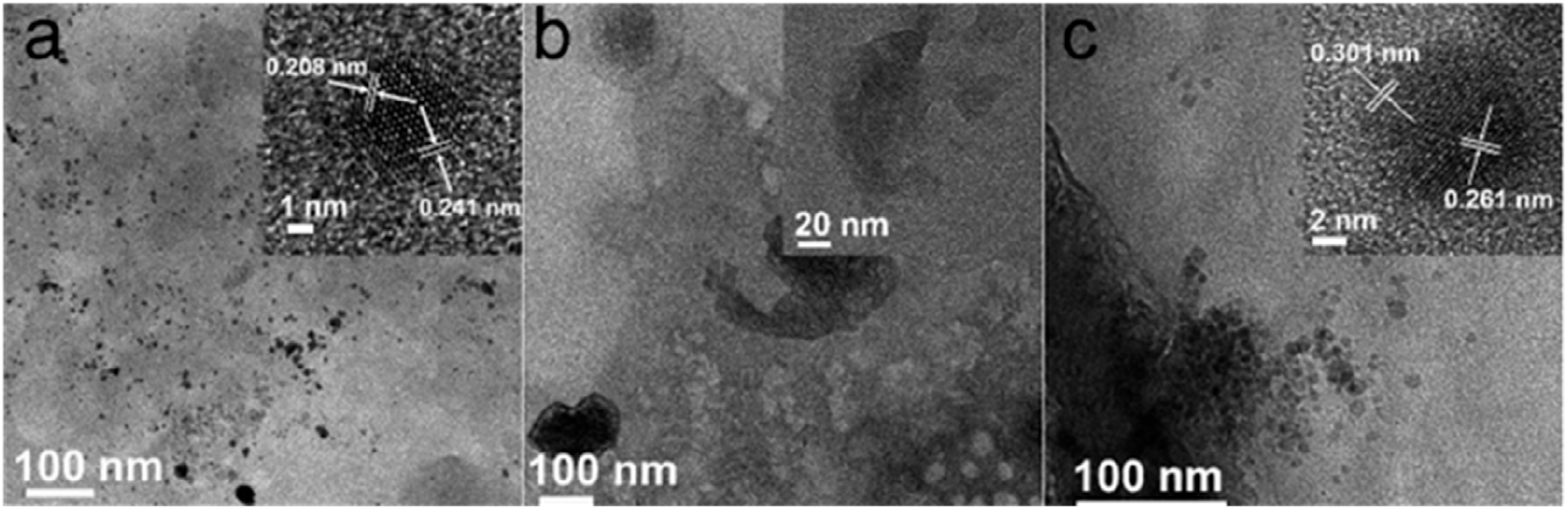
TEM phrotographs of **(A)** CoPor@G_P_F, **(B)** CoPht@G_P_F, and **(C)** CoVB_12_@G_P_F. Insets are higher magnification images, which demonstrate either crystal lattice or enlarged details.

The sample of Co-Por sonicated off from the CoPor@G_P_F surface has a size distribution from 1 nm to ca. 15 nm, which can be seen in the TEM picture of Figure 5. We found that smaller-sized particles of Co-Por, typically 1–5 nm in size, exhibited single crystal crystallinity. The crystal lattice was measured as ca. 0.208 nm and 0.241 nm for one particle exampled in a high-resolution TEM image (Figure 5). When the particle size is bigger than ca. 5 nm, the polycrystal structure of the particles was observed, which shows random crystallographic orientations. The broad size distribution and polycrystallinity of the Co-Por particles reflect that the nucleation and crystal growth process of Co-Por when deposited on graphite were not homogeneous.

For Co-Pht sample, the size of the particles is not homogeneous and is bigger than 200 nm. In addition, we did not observe a clear crystal lattice of Co-Pht under TEM (Figure 5B), indicating its low crystallinity. In comparison, Co-Pht had been grown on a KCl {001} substrate in previous work by repeated vacuum deposition and thermal treatment (Yanagiya et al., 2004). As a result, ellipsoid-shaped particles with lengths in micrometer and crystal planes were obtained. The difference in crystallinity should be due to the different conditions of crystal growth.

The Co-VB_12_ sample has a broad size distribution, from around 10 nm to around 200 nm. However, both small and large particles were observed with well-developed single crystal structures. Taking one particle as an example, the lattice spacing was measured as 0.301 nm and 0.261 nm as shown in Figure 5C. This indicates that although the nucleation of Co-VB_12_ might not be homogeneous, the crystal growth had occurred.

### 2.3 Electrocatalytic H_2_-production

**FIGURE 6:**
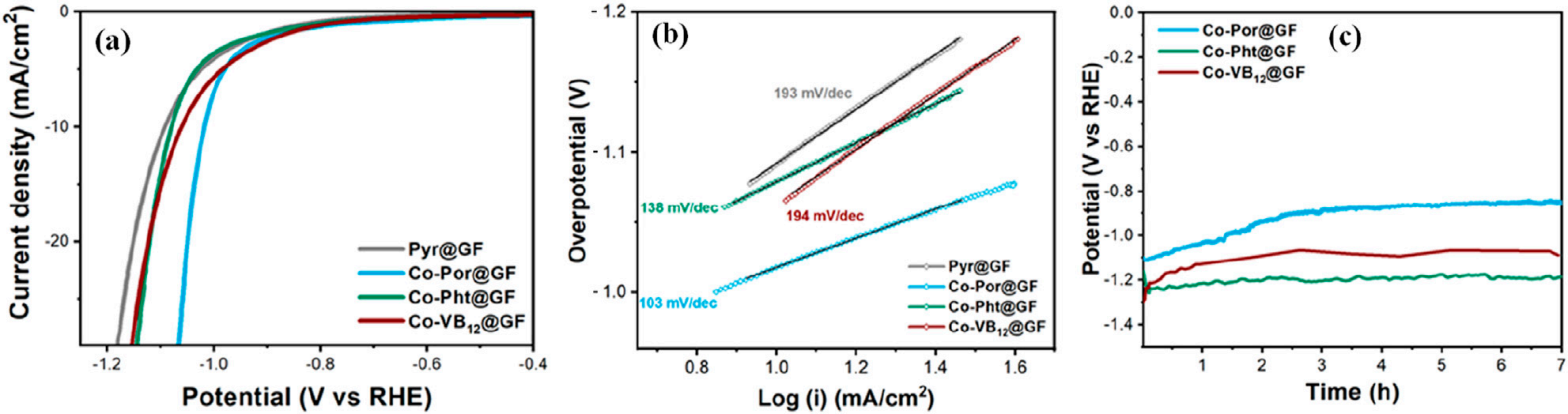
LSV measurement **(A)** and corresponding Tafel plots **(B)** for graphite foil (grey), CoPor@G_P_F (blue), CoPth@G_P_F (green), and CoVB_12_@G_P_F (maroon) in pH 7.1 phosphate buffer (0.1 M) solution purged with N_2_. Ag/AgCl (3 M) was used as the reference electrode and the graphite bar was used as the counter electrode. **(C)** Chronopotentiometric response at a current density of 10 mA/cm^2^ over a period of 7 h.

The electrocatalytic hydrogen production was evaluated using linear sweep voltammetry (LSV) (Figure 6). For CoPor@G_P_F sample, water reduction starts at −743 mV vs. RHE at 2.5 mA/cm^2^, which is 85 and 90 mV lower overpotential than that of Co-Pht@G_P_F and CoVB_12_@G_P_F, respectively. Although Co-Pht (before being anchored on the graphite surface) showed the best water reduction features in homogeneous conditions, it comes out to be less efficient after anchoring onto G_P_F among the samples studied. This can be due to the inefficient charge transfer from the Co-Pht to G_P_F, due to the intrinsic structural limitations (El-Khouly et al., 2004). Previously in XPS analysis, a higher concentration of cobalt was detected in CoPht@GPF sample in comparison with the other, possessing a higher ratio of cobalt active sites. Therefore, the active mass of the molecular catalyst framework alone does not dictate electrocatalytic performance. Other factors, such as the anchoring efficiency of the ligand frameworks and the optimal ligand framework-to-graphite ratio, likely play a crucial role. Interestingly, CoVB_12_@G_P_F showed very similar initial water reduction behavior with CoPor@G_P_F until −1 V, followed by a clear deviation in the electrochemical response (Figure 6A). CoVB_12_@G_P_F started exhibiting similar current density features as of CoPht@G_P_F from −1.12 V onward negative potentials. The steric restrictions and hydrophobic moieties around the Co-center in vitamin B_12_ are expected to be the reasons behind the slow kinetic water reduction features of CoVB_12_@G_P_F (Figure 1 and Supplementary Figure S3) (Giedyk et al., 2015). Moreover, the large size of the VB_12_ does not allow higher catalyst loading on G_P_F, resulting lower Co: surface area ratio in CoVB_12_@G_P_F in comparison to that of CoPor@G_P_F and CoPht@G_P_F, which also impacts in the water reduction reactivity. The better electrocatalytic features and catalyst loading were confirmed by the Tafel plots analysis, which shows a much lower slope value (103 mV/dec) for CoPor@G_P_F, followed by CoPht@G_P_F and CoVB_12_@G_P_F with values of 138 mV/dec and 194 mv/dec, respectively (Figure 6B). It is widely accepted that the value of the Tafel slope has a close relationship with the HER mechanism (Mahmood et al., 2018). The values obtained from the samples in this work are associated with a Volmer-limited reaction, i.e., the HER limited by the hydrogen adsorption reaction to form M-H_ads_ intermediates (Li F. et al., 2019). All of these three electrodes were tested over a period of 7 h under water reduction conditions. TOF values were calculated (see Supplementary Material) at a current density of 10 mA/cm^2^, where steady hydrogen production was confirmed using an H_2_ needle sensor. It showed Co-porphyrin containing electrode a TOF value of 0.45 s^−1^ at 870 mV vs. RHE, whereas for Co-phthalocyanine and Vitamin-B_12_ containing electrodes showed TOF value of 0.37 and 0.4 s^−1^ at 1.22 V and 1.15 V (vs. RHE), respectively.

But firstly, in order to understand the potential reasons behind the contradictory behaviour between homogeneous and heterogeneous electrocatalytic tests, we performed impedance measurements (EIS) at different potentials to analyse the different contributions in the electrocatalytic process. Supplementary Figure S4A shows the Nyquist plot, comparing the impedance response of all three electrocatalysts at −1.0 V. The curves were zero-corrected to better visualize the differences on the electrode mechanisms. It is clear that CoPor@G_P_F shows the lowest total resistance, as observed by the diameter of the semicircle in Nyquist plot. As expected for CoPor@G_P_F sample, at larger overpotential, a reduction in the impedance magnitude was observed, as shown in Supplementary Figure S4B. To further distinguish the different contributions during electrocatalysis the impedance curves were fitted using the equivalent circuit presented in Supplementary Figure S4C. The circuit consist of a two-time constant wherein we proposed a first time constant (CPE1 and R1) is associated to the electrolyte-electrode interface and a second time constant (R2 and CPE2) associated to the interface between the molecular active phase catalyst and the conductive substrate (G_P_F). It was not possible to distinguish between different contributions in case of CoVB_12_@G_P_F, but the resistance values for R1 and R2 of CoPht@G_P_F and CoPor@G_P_F are plotted in Supplementary Figure S4D. From the resistance values it can be observed that CoPht@G_P_F shows slightly lower resistance to the charge transfer that is driving the catalytic reaction, well correlating with the results obtained by homogeneous study. On the other hand, a significant difference is observed in R2 values, with lower resistance values for CoPor@G_P_F in the overpotential windows tested, which indicates a more favoured conduction of charges through the ligand in case of CoPor@G_P_F, compared to CoPht@G_P_F. It can be associated to the poor distribution of the molecular catalysts on the surface of the conductive substrate. The large agglomerates make the charge transfer from the conductive support to the metal centre significantly more difficult.

The potential vs. time plot (Figure 6C) clearly shows that CoPor@G_P_F improves its reactivity (lower initial potential required to achieve 10 mA/cm^2^ current) with time, which is directly related to overpotential. Almost 220 mV improvement of the initial potential was observed for hydrogen evolution reaction (HER) during first 3 h. It is followed by stable current flow and hydrogen production for the next 4 h. The electrolyte solutions after these 7 h electrolysis experiments were investigated by 500 MHz ^1^H NMR. No evidence of metal leaching or ligand decomposition was observed. The improvement in overpotential can be attributed to the slow coordination of the water molecule and formation of a hydrogen-bonding network around the cobalt center, which is most prominent with CoPor (Okamoto et al., 1975). Water coordinated Co(OH_2_)-Por@G_P_F is expected to be a more efficient cathode than CoPor@G_P_F itself due to easier proton transfer from the activated water molecule at the Co-OH_2_ center (Cheng et al., 2022). This activation by water coordination can be corroborated with the highest value of oxygen percentage (O % ∼ 9.3%) after the impregnation (Supplementary Table S2) with CoPor@G_P_F. Furthermore, the improvement in overpotential could also be related to the presence of reduced Co^0^ species, which may emerge under reducing conditions and have been shown to be active in electrochemically relevant reactions (Gupta et al., 2023; Zhang et al., 2023). Electrochemical impedance spectroscopy (EIS) measurements further highlight the better performance of CoPor@G_P_F. The superior conductivity observed in this sample facilitates efficient electron transfer to the active Co-OH_2_ centers, promoting the HER process. Additionally, transmission electron microscopy (TEM) reveals smaller particle sizes in CoPor@G_P_F compared to the other samples. This reduced particle size translates into a larger active surface area, providing more active sites for the HER reaction to occur. Regarding thermal stability, TGA also reveals significant mass losses in CoPor@G_P_F between 250°C and 450°C, reaching approximately 5%, while the remaining samples exhibit only around 1% mass loss. This observation confirms the stability of CoPor@G_P_F at temperatures below 250°C. The mass loss at higher temperatures is likely associated with the decomposition of weakly anchored porphyrin groups on the graphite surface, potentially contributing to the enhanced electron transfer at the Co-OH_2_ centers and also enhancing the electrocatalytic activity.

Although all three electrodes showed stable features under water reduction (hydrogen production) conditions and continuous hydrogen evolution, no such distinct activation features were observed in the case of CoPht@G_P_F and CoVB_12_@G_P_F, implying that the formation of Co(OH_2_) type species and corresponding improved reactivity is much more prominent in case of CoPor@G_P_F which is proving out to be the best water reducing electrode among all three molecular electrodes.

### 2.4 DFT calculations

**FIGURE 7:**
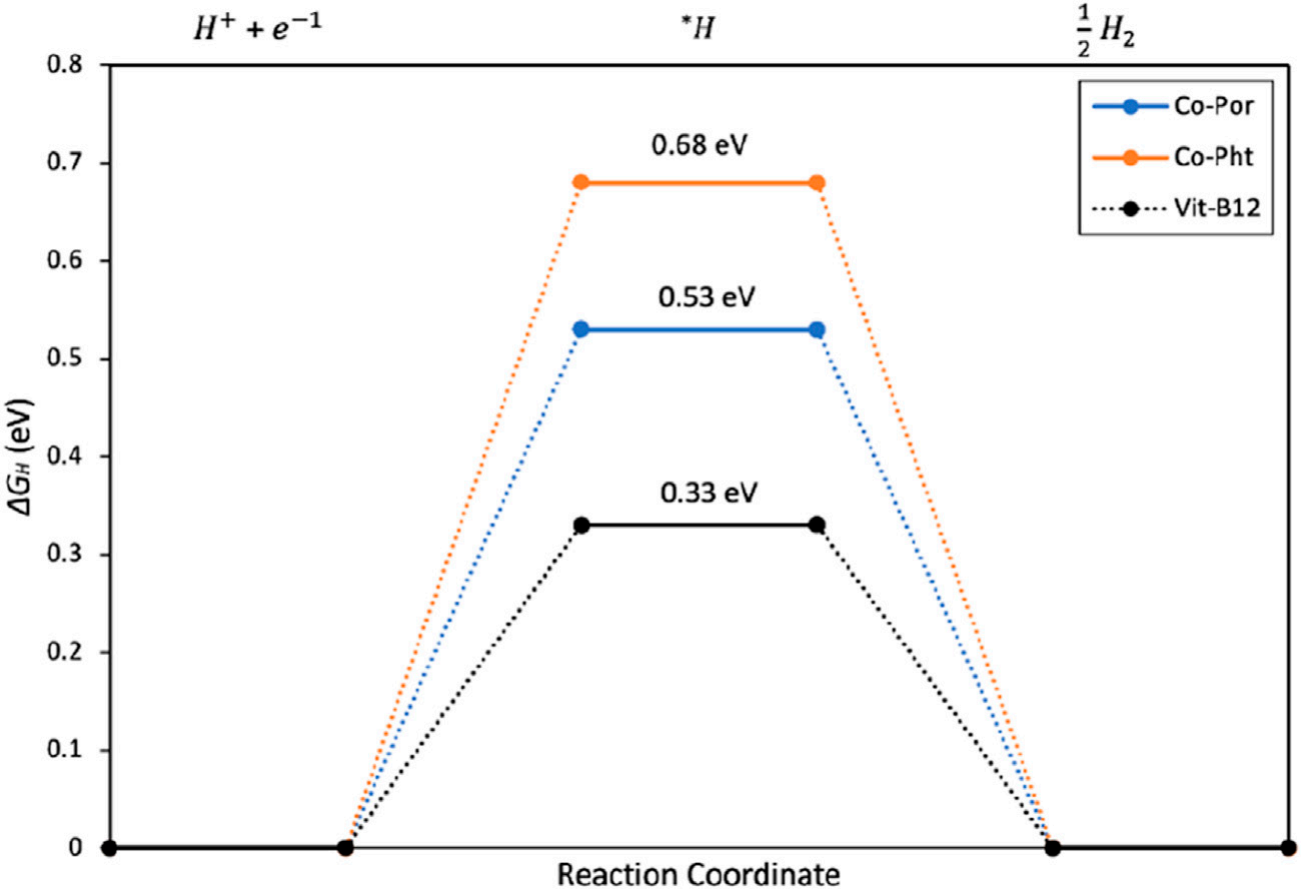
DFT calculated Reaction energy diagram for H_2_ production using Co-Por, Co-Pht, and Vit-B_12_ as electrocatalysts.

We carried out DFT-based free energy calculations of H adsorption at the Co site in the unsupported catalysts to understand the difference in activity between these three Co-based electrocatalysts in H_2_ production (Figure 7). The model structures utilized in these calculations are the same as shown in Figure 1 and Supplementary Figure S5. However, for VB_12_, we removed the -CN moiety and attached H instead. All the structures were geometrically optimized to minimize forces acting on the atoms (see Supplementary Material for details).

It is generally accepted that the free energy changes associated with H adsorption $∆G_{H^{*}}$ is a key descriptor for HER catalysts (Ju et al., 2017). A HER catalyst is expected to perform well when the free energy value is close to thermoneutral, i.e., $∆G_{H^{*}}\approx 0$ . The results indicate a more optimal hydrogen adsorption for the Vit-B_12_ sample, with a $∆G_{H^{*}}$ value of 0.33 eV, followed by the Co-Pht and Co-Por samples, with values of 0.53 and 0.68 eV, respectively. These findings suggest that Vit-B_12_ has the best performance for the hydrogen evolution reaction (HER). However, experimental results have shown that the most electroactive sample is CoPor@G_P_F. This discrepancy highlights the influence of molecule adsorption on the graphite sheet, which alters electron transfer, as demonstrated by EIS, thereby modifying the electrocatalytic activity towards HER.

## 3 Experimental and methods

### 3.1 Materials

All three catalysts [Co(II)tetrakis (4-methoxyphenyl)-porphyrin, Cobalt (II) phthalocyanine, and Vitamin B_12_] that are used in this project were commercially available from Merck. They were received as pure (∼99%) compounds and used as catalysts without any additional purification. Prior to the catalysts’ anchoring, the graphite foil surface was structurally modified following our reported procedure (Das et al., 2023).

### 3.2 Methods

The surfaces of the pyridine-modified graphite and the modified graphite with Co-complexes catalysts were investigated by scanning electron microscopy (SEM) using Zeiss Gemini Ultra 55 microscope. Transmission electron microscopy (TEM) analysis was performed using an aberration-corrected Themis Z instrument from Thermo Fisher. TEM was operated at 300 kV in Scanning TEM (STEM) mode. The samples were prepared by detaching the catalyst from the graphite substrate by sonication of CoPor@G_P_F, CoPht@G_P_F, and CoVB_12_@G_P_F in 1 M aqueous KOH for 30 min and dispersed onto a thin carbon support film.

The surface of the electrocatalysts was also examined by X-ray photoelectron spectroscopy (XPS) using a VG-Microtech Multilab 3,000 spectrometer (VG Scientific, Sussex) equipped with a hemispherical electron analyzer with nine channeltrons and an X-ray source with Al radiation. The binding energy of the C 1s peak at 284.6 was taken as an internal standard. Thermogravimetric analysis (TGA) (under N_2_ with flow of 5 mL/min in balance and 45 mL/min in furnace) was performed using a TGA Q500 equipment (TA Instrument) under a nitrogen atmosphere to determine weight losses due to the elimination of surface functionalities.

Redox response in the homogeneous condition (Figure 2A) was investigated using a glassy carbon electrode (3 mm diameter) as the working electrode, Ag/AgCl/Cl (sat.) as the reference electrode, and graphite as the counter electrode in 0.1 M phosphate buffer solution (pH 7.1) as electrolyte. Electrocatalytic properties of the designed electrodes were studied in an electrochemical workstation connected to a Potentiostat/Galvanostat/ZRA Gamry Interface 1010E. All the potentials were converted into a reversible hydrogen electrode (RHE) following this equation:

$$E_{RHE}=0.1976+E_{Ag|AgCl}+0.059pH$$

The hydrogen evolution reaction (HER) activity was studied by linear sweep voltammetry in the potential window from 0 to −1.4 V vs. RHE at scan rate of 100 mV/s, to determine the overpotentials and Tafel slopes. Electrochemical impedance spectroscopy (EIS) was employed to study the contribution of the structural features of the catalysts. It was conducted under potentiostatic conditions in a potential window between −0.8 and −1.0 V vs RHE with a signal amplitude 10 mV rms in the frequency range between 100 kHz and 100 mHz. All the experiments were performed after purging the electrolyte with N_2_ for 15 min.

Chronopotentiometric experiments were performed under a constant current of 10 mA/cm^2^ to study the stability of the HER catalyst and evaluate the H_2_ production performance over time. Complementary, hydrogen was detected during chronopotentiometry using a H_2_ needle sensor (2.1 × 80 mm) connected to an H2 UniAmp unit (Unisense).

### 3.3 Computational methods

All structural relaxation calculations were performed using a plane-wave basis set as implemented in the VASP package until the residual forces on atoms were less than 0.03 eV/ $\overset{{}^{\circ}}{A}$ (Kresse and Furthmüller, 1996). We employed the Projector Augmented Wave (PAW) method to describe the core electrons and the Perdew Burke Ernzerhof exchange-correlation (XC) functional (Perdew et al., 1996; Joubert, 1999). The kinetic energy cut-off for the wave function and charge density was set to 500 eV, and a gamma-point k-grid was used. A vacuum region greater than 10 $\overset{{}^{\circ}}{A}$ was used to avoid interaction between neighbouring images.

The hydrogen adsorption energy is first calculated as:

∆EH*=Ecatalyst+H*−Ecatalyst−12EH2

where, $E_{\text{catalyst}+H^{*}}$ is the total energy of the catalyst with an H atom attached, $E_{\text{catalyst}}$ is the total energy of the same system with no adsorbed hydrogen and $E_{H_{2}}$ is the energy of a hydrogen molecule in the gas phase. The free energy of hydrogen adsorption is then calculated as $∆G_{H^{*}}=∆E_{H^{*}}+0.24\text{ eV}$ eV, to account for the changes in the zero-point energy and the entropy between the adsorbed state and the gas phase of hydrogen.

## 4 Conclusion

Three new water-reduction molecular electrodes were synthesized utilizing environmentally benign and readily available cobalt containing catalysts, with the idea of producing cost effective and green hydrogen through water reduction at neutral pH. Among these different catalysts’ frameworks (porphyrin, phthalocyanine, and corrin) and under the used reaction conditions, the Co-Porphyrin on the modified graphite foil surface showed the best electrocatalytic performance. Molecular level activation due to coordination of the water molecule is anticipated and supported by spectroscopic techniques as the possible reason behind the enhanced activity. Although in homogeneous conditions, Co-Phthalocyanine moiety showed better water reductive current, decreased reactivity after anchoring on the graphite foil surface was observed. This is attributed to the restricted electron transfer due to the spatial orientation and supported by the EIS study. In agreement with the experimental results (e.g., XPS, EIS, Chronopotentiometry), DFT based free energy calculations show better (85 mV lower overpotential) hydrogen production features of Co-Porphyrin than that of the Co-Phthalocyanine moiety.

## Data Availability

The original contributions presented in the study are included in the article/Supplementary Material, further inquiries can be directed to the corresponding author.
