# Supplementary material for: Co-complexes on modified graphite surface for steady green hydrogen production from water at neutral pH
Source: Front Chem. 2024 Sep 25;12:1469804. doi: 10.3389/fchem.2024.1469804 (PMC11472121; doi:10.3389/fchem.2024.1469804)
Supplement: Supplementary file 1 [file DataSheet1.docx]

***Supporting information for***

**Co-complexes on Modified Graphite Surface for Steady Green Hydrogen Production from Water at Neutral pH**

*Esteban A. Toledo-Carrillo^a^* ^†^, *Mario García Rodríguez^b^* ^†^*,*  *Emilia Morallón^b^, Diego Cazorla-Amorós^c^, Fei Ye^a^, Varun Kundi^d^, Priyank V. Kumar^d^, Oscar Verho^e^, Joydeep Dutta^a^, Björn Åkermark^f^, Biswanath Das^* f^*

^a^ Department of Applied Physics, KTH Royal Institute of Technology, Hannes Alfvéns väg 12, 114 19 Stockholm, Sweden.

^b^ Departamento de Química Física e Instituto Universitario de Materiales, Universidad de Alicante, Ap. 99, E-03080, Alicante, Spain.

^c^ Departamento de Química Inorgánica e Instituto Universitario de Materiales, Universidad de Alicante, Ap. 99, E-03080, Alicante, Spain.

^d^ School of Chemical Engineering, University of New South Wales, Sydney 2052, Australia.

^e^ Department of Medicinal Chemistry, Biomediciniskt Centrum BMC, Uppsala University, SE-75123 Uppsala, Sweden.

^f*^ Department of Organic Chemistry, Arrhenius Laboratory Stockholm University, Svante Arrhenius Väg 16C, 10691 Stockholm, Sweden. [*das.biswanath85@gmail.com/biswanath.das@su.se*](mailto:das.biswanath85@gmail.com/biswanath.das@su.se)

^†^ Contributed equally to this project.

**Characterization:**

**Figure S1.** Post- and pre-impregnated samples in 0.1 M phosphate electrolyte for a) N 1s, b) Co 2p, c) C 1s, and d) O 1s.

**Table S1**. XPS deconvolution parameters.

| **Sample** | **Element** | **Species** | **Peak Position (eV)** | **Area (a. u.)** | **FWHM (eV)** |
| --- | --- | --- | --- | --- | --- |
| **CoPor@G_P_F** | **C** | C sp^2^ | 284.57 | 0.71 | 0.70 |
|  |  | C-OH | 285.36 | 0.14 | 1.08 |
|  |  | O-C=O | 286.30 | 0.06 | 1.00 |
|  | **N** | 2-Methylbenzimidazole | - | - | - |
|  |  | N-Pyridinic | 398.95 | 0.44 | 1.30 |
|  |  | CN + Amide | - | - | - |
|  |  | N-Pyrrolic | 400.15 | 1.37 | 1.60 |
|  |  | N^+^ | 401.35 | 0.21 | 1.20 |
|  | **O** | C=O | 531.30 | 0.27 | 1.40 |
|  |  | C-O | 532.60 | 2.02 | 2.00 |
|  |  | C-OH | 533.90 | 0.42 | 1.30 |
| **CoPht@G_P_F** | **C** | C sp^2^ | 284.65 | 0.74 | 0.72 |
|  |  | C-OH | 284.40 | 0.13 | 0.96 |
|  |  | O-C=O | 286.39 | 0.10 | 1.62 |
|  | **N** | 2-Methylbenzimidazole | - | - | - |
|  |  | N-Pyridinic | 398.89 | 1.20 | 1.23 |
|  |  | CN + Amide | - | - | - |
|  |  | N-Pyrrolic | 400.25 | 0.27 | 1.20 |
|  |  | N+ | 401.10 | 0.07 | 0.70 |
|  | **O** | C=O | 531.30 | 0.30 | 1.44 |
|  |  | C-O | 532.55 | 1.80 | 2.00 |
|  |  | C-OH | 534.00 | 0.78 | 2.00 |
|  | **Co** | Co^2+^ | 780.60 | 2.26 | 0.90 |
|  |  | Sat. | 782.50 | 1.51 | 1.30 |
| **CoVB_12_@G_P_F** | **C** | C sp^2^ | 284.57 | 0.74 | 0.71 |
|  |  | C-OH | 285.42 | 0.11 | 1.05 |
|  |  | O-C=O | 286.39 | 0.06 | 1.10 |
|  | **N** | 2-Methylbenzimidazole | 398.00 | 0.19 | 0.90 |
|  |  | N-Pyridinic | 398.70 | 0.52 | 1.04 |
|  |  | CN + Amide | 399.40 | 0.70 | 0.80 |
|  |  | N-Pyrrolic | 400.31 | 0.62 | 0.90 |
|  |  | N+ | 401.20 | 0.46 | 1.25 |
|  | **O** | C=O | 531.30 | 0.41 | 1.70 |
|  |  | C-O | 532.55 | 1.78 | 2.00 |
|  |  | C-OH | 533.90 | 0.58 | 1.70 |

**Table S2.** Mass and atomic percentage of all samples before and after impregnation with 0.1 M phosphate buffer electrolyte.

|  |  |  | **Element** | | | | |
| --- | --- | --- | --- | --- | --- | --- | --- |
|  | **Sample** | **%** | **C** | **O** | **N** | **Co** | **F** |
| **Pre-impregnation** | **CoPor@G_P_F** | atomic | 95.27 | 2.51 | 1.17 | 0.10 | 0.95 |
|  |  | mass | 94.15 | 3.31 | 1.35 | 0.48 | 0.70 |
|  | **CoPht@G_P_F** | atomic | 95.6 | 2.23 | 1.76 | 0.32 | 0.09 |
|  |  | mass | 93.41 | 2.91 | 2.01 | 1.53 | 0.14 |
|  | **CoVB_12_@G_P_F** | atomic | 97.69 | 1.54 | 0.52 | 0.10 | 0.18 |
|  |  | mass | 96.6 | 2.03 | 0.6 | 0.49 | 0.28 |
| **Post-impregnation** | **CoPor@G_P_F** | atomic | 89.91 | 7.23 | 1.98 | 0.07 | 0.89 |
|  |  | mass | 86.87 | 9.31 | 2.12 | 0.33 | 1.36 |
|  | **CoPht@G_P_F** | atomic | 91.6 | 5.45 | 2.53 | 0.36 | 0.06 |
|  |  | mass | 88.35 | 7.00 | 2.84 | 1.69 | 0.09 |
|  | **CoVB_12_@G_P_F** | atomic | 92.46 | 5.62 | 1.76 | 0.08 | 0.10 |
|  |  | mass | 90.16 | 7.31 | 2.00 | 0.38 | 0.15 |

**Table S3.** The ratio of the species detected in the deconvolution for each of the samples.

|  |  | **N-pyridinic** | **N-Pyrrolic/**  **N-Co** | **N^+^** | **CN/**  **Amide** | **2-methylbenzimi-dazole** | **C=O** | **C-O** | **C-OH** |
| --- | --- | --- | --- | --- | --- | --- | --- | --- | --- |
| **Pre-impregnation** | **CoPor@G_P_F** | 21.8 | 68.0 | 10.2 | - | - | 9.8 | 74.6 | 15.6 |
|  | **CoPht@G_P_F** | 72.2 | 23.4 | 4.4 | - | - | 10.4 | 62.5 | 27.1 |
|  | **CoVB_12_@G_P_F** | 14.8 | 28.2 | 9.0 | 40.0 | 8.0 | 14.7 | 64.2 | 21.1 |
| **Post-impregnation** | **CoPor@G_P_F** | 34.6 | 52.7 | 12.7 | - | - | 29.1 | 59.1 | 11.0 |
|  | **CoPht@G_P_F** | 61.3 | 32.7 | 6.0 | - | - | 12.8 | 64.2 | 23.0 |
|  | **CoVB_12_@G_P_F** | 14.0 | 50.3 | 8.8 | 20.9 | 6.0 | 15.8 | 63.4 | 20.8 |

**Figure S2.** XPS deconvolution spectrum of the pre- and post-impregnated with 0.1 M buffer phosphate electrolyte.

**Calculation of turnover frequency (TOF):**

TOF is calculated using from the potential vs time plot at 10mA/cm^2^ stable current density [(i.e., 0.87 V, 1.22 V, and 1.15 V (vs RHE), figure 4c] when continuous H_2_ production was monitored by H_2_ needle sensor (2.1 x 80 mm) connected to an H_2_ UniAmp unit. The calculation was done following a previously reported procedure (*J. Mater. Chem. A, 2023,11, 13331-133409*).

10 mA = 0.01 Coulomb/s

96500 coulomb = 1 mole of electrons

0.01 coulomb/s = 1.036 × 10^-7^ mole of electrons/s ≡ 5.18 × 10^-8^ moles of hydrogen production/s

To minimize the error of calculating the catalyst amount on GpF, the same procedure was repeated five times and an average was taken into consideration. The remains of the Co-Por, Co-Pht, and Co-VB_12_ in aqueous and ethanol solution after washing were also considered while calculating the correct amount of catalysts attached to GpF.

A total amount of 1.8 mg, 1.6 mg, and 2.6 mg of Co-Por, Co-Pht, and Co-VB_12_ respectively lost from the stock solution was observed after anchoring them onto five batches of 2 cm x 2 cm GpF electrode. This means one 1 cm x 1 cm GpF electrode contains 0.9 x 10^-4^, 0.8 x 10^-4^, and 1.3 x 10^-4^ mg of Co-Por, Co-Pht, and Co-VB_12_ respectively, i.e., 1.14 x 10^-7^, 1.4 x 10^-7^, and 1.28 x 10^-7^ moles of Co-Por, Co-Pht, and Co-VB_12_ respectively. Corresponding TOF values are:

5.18 × 10^-8^ / 1.14 x 10^-7^ = 0.45 s^-1^ for CoPor@G_P_F at 0.87 V vs RHE

5.18 × 10^-8^ / 1.4 x 10^-7^ = 0.37 s^-1^ for CoPht@G_P_F at 1.22 V vs RHE

5.18 × 10^-8^ / 1.28 x 10^-7^ = 0.4 s^-1^ for CoVB_12_@G_P_F at 1.15 V vs RHE


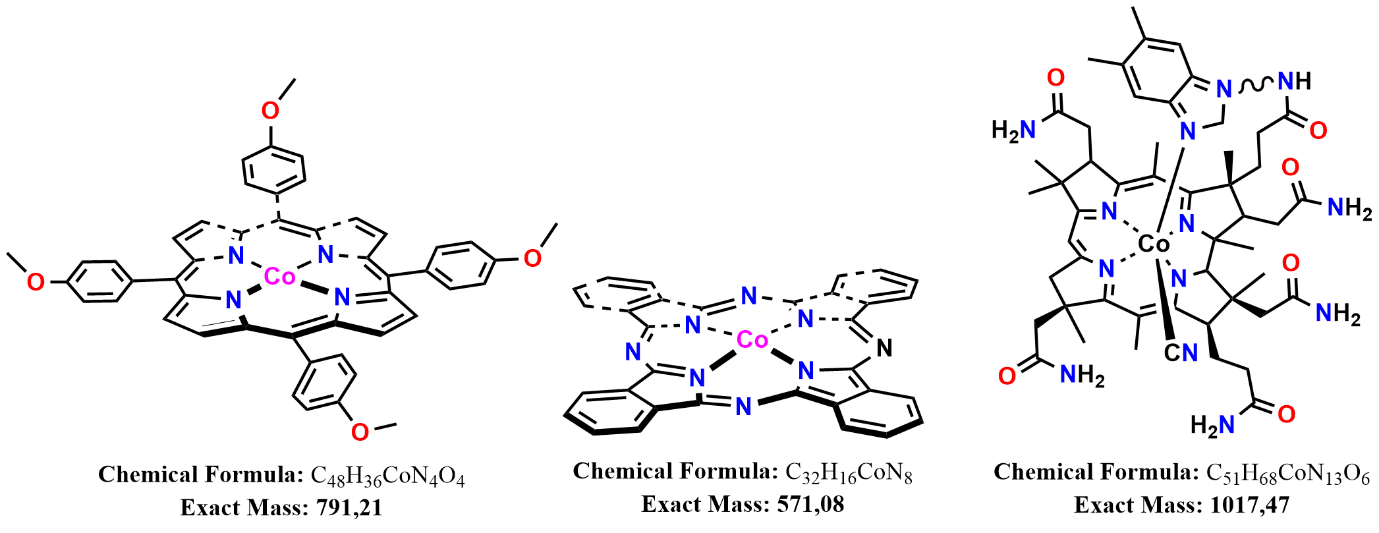


**Figure S3.** The molecular catalysts tested in this project, and corresponding molecular weights used for TOF calculations.

**
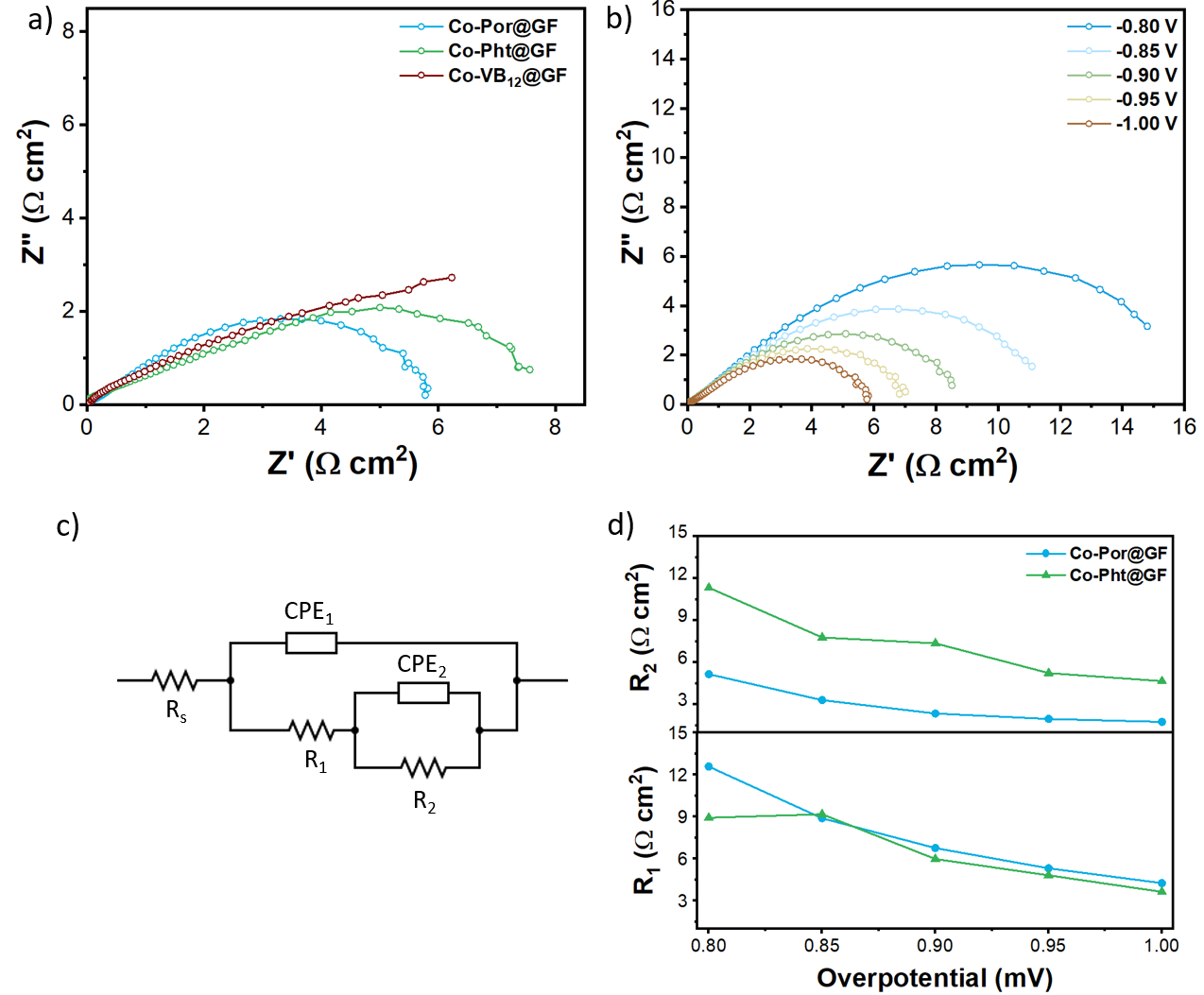
**

**Figure S4.** Impedance measurements: Nyquist plot showing the impedance response of (a) three electrodes CoPor@G_P_F, CoPht@G_P_F, and CoVB_12_@G_P_F at 1.0 V overpotential and (b) impedance of CoPor@G_P_F at different overpotential. (c) Equivalent circuit for fitting and (d) obtained values for R1 and R2 from the fitting at different overpotentials.

**DFT calculated optimized geometries:**


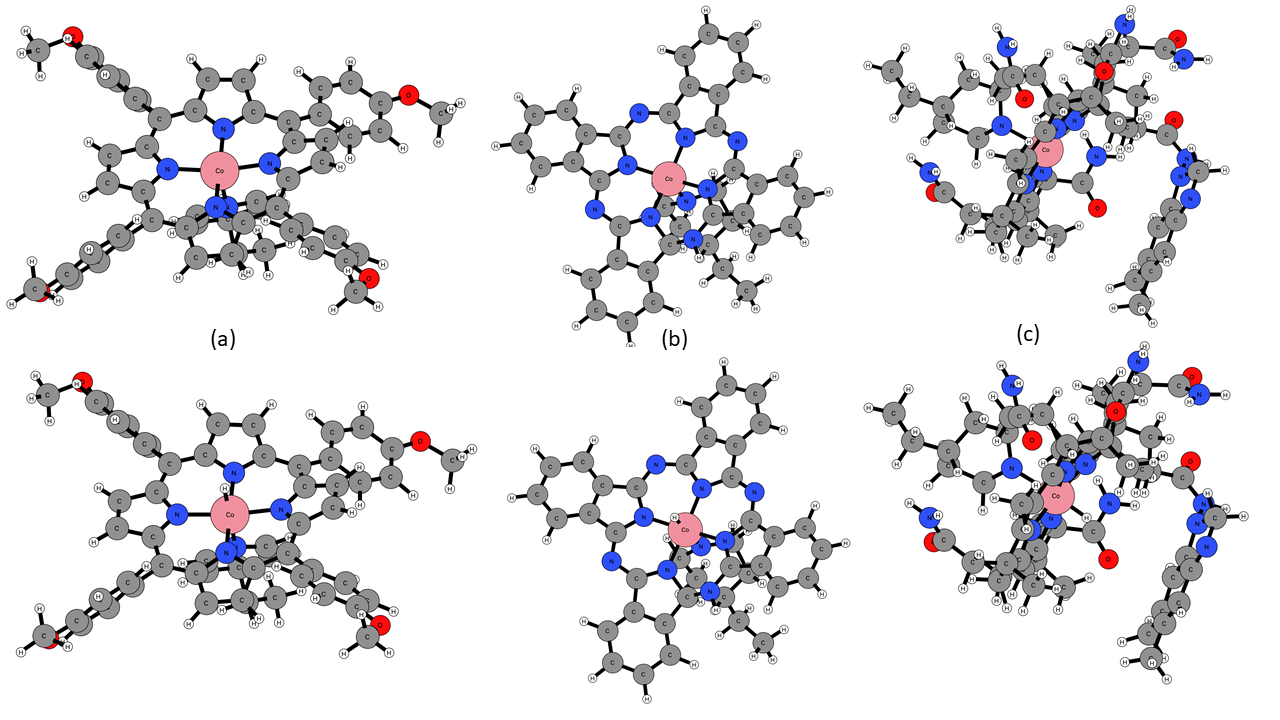


**Figure S5.** DFT optimized structures of the molecular electrocatalysts (upper row) and corresponding structures after H-adsorption (lower row): (a) Cobalt (II) tetrakis(4-methoxyphenyl) -porphyrin (CoPor), (b) Cobalt (II) phthalocyanine (CoPht), and (c) Vitamin B_12_ (CoVB_12_).

**Cartesian coordinates of the optimized structures:**

Cobalt (II) tetrakis(4-methoxyphenyl) -porphyrin (CoPor):

111 (no. of atoms)

C Co H N O

C 3.147895 5.680878 14.732051

C 4.785230 7.343411 13.101355

C 4.905692 5.791192 2.602425

C 5.195203 6.867031 14.353743

C 5.700829 8.012236 12.284254

C 6.192948 7.152560 4.716545

C 6.514346 7.090642 14.777898

C 6.895900 6.348311 3.808984

C 6.884745 7.791749 5.747951

C 7.034922 8.214693 12.673188

C 7.414775 7.746058 13.946393

C 7.525707 7.093660 10.026705

C 7.771939 6.980710 8.692611

C 8.285204 6.196710 3.952533

C 8.011516 8.851629 11.748328

C 8.272703 7.657797 5.906091

C 8.177779 8.299999 10.471236

C 8.588967 8.106954 8.325154

C 8.958698 6.842893 4.986563

C 8.595830 10.589188 13.451487

C 8.747441 9.963634 12.167052

C 8.942829 8.406333 7.008575

C 9.653285 11.435542 13.607126

C 9.790357 9.470099 6.691398

C 10.174586 9.801469 5.344685

C 10.430018 11.366607 12.396798

C 11.060796 10.832559 5.429237

C 11.180681 11.164142 6.822273

C 11.621476 12.098943 12.187785

C 11.914765 12.492771 10.861459

C 11.957937 12.215427 7.305340

C 12.003275 12.552328 8.657606

C 12.409458 11.578128 14.508559

C 12.512243 9.092076 10.979091

C 12.488189 12.398956 13.357992

C 12.460656 8.528951 8.720772

C 12.307955 13.833981 5.371416

C 12.638910 13.679051 10.487278

C 12.680768 13.722069 9.130267

C 12.825428 12.997848 6.375386

C 13.183148 11.783904 15.652274

C 13.156253 14.594624 4.562508

C 13.499986 13.391658 13.409417

C 13.851544 8.885408 11.061851

C 13.797953 8.295912 8.693767

C 14.126993 12.817445 15.678148

C 14.292153 13.599744 14.527055

C 14.221522 12.939053 6.538254

C 14.680032 8.437740 9.897156

C 14.541274 14.526800 4.748693

C 15.075814 13.691634 5.733247

C 14.718339 12.426275 17.962141

C 15.460755 7.120613 10.186268

C 15.715146 14.822195 2.714033

C 16.529926 7.253408 11.270553

Co 10.302016 10.258590 9.582369

H 2.697743 5.125495 15.563154

H 2.467427 6.494405 14.431411

H 3.763883 7.199529 12.751899

H 4.593752 6.840507 2.473987

H 3.305467 5.001332 13.877439

H 4.660980 5.225606 1.697403

H 5.116477 7.291803 4.629191

H 4.372176 5.353871 3.461743

H 5.368667 8.383530 11.315005

H 6.824294 6.712652 15.752279

H 6.330197 8.424578 6.443600

H 6.951712 6.425987 10.658705

H 7.440168 6.207353 8.008132

H 8.447451 7.873512 14.272100

H 7.774705 10.410914 14.136993

H 8.818126 5.561982 3.243373

H 10.037515 6.710557 5.085128

H 9.860634 12.103113 14.435928

H 9.809230 9.299624 4.455523

H 11.227051 13.912427 5.243374

H 11.734085 10.725299 14.507821

H 11.577599 11.342813 4.623035

H 11.954275 9.362954 11.873305

H 11.864172 8.351095 7.827305

H 13.056977 11.112500 16.500721

H 12.749667 15.270441 3.808048

H 12.947733 14.480039 11.141365

H 13.089727 14.503795 8.500373

H 13.707256 14.026072 12.560339

H 14.323946 8.978692 12.041953

H 14.236788 7.900142 7.773432

H 13.698114 12.603590 18.339490

H 14.635996 12.293037 7.313858

H 15.054988 14.379147 14.524300

H 15.470040 9.197715 9.691005

H 14.812633 14.710639 2.089807

H 14.732504 6.339648 10.459127

H 15.448909 12.832664 18.669518

H 16.157751 13.652732 5.866843

H 14.884149 11.342391 17.852076

H 15.930581 6.796250 9.242149

H 16.094069 7.519432 12.244220

H 16.388501 15.550925 2.248226

H 16.224030 13.846146 2.787745

H 17.266843 8.030035 11.012249

H 17.073276 6.307085 11.404996

N 8.890237 8.878480 9.435774

N 9.818255 10.532213 11.477265

N 10.399011 10.324266 7.601744

N 11.451278 11.845454 9.719708

N 11.738956 8.895358 9.847913

O 4.392196 6.178936 15.226259

O 6.319826 5.679178 2.760656

O 14.940366 13.121768 16.734500

O 15.394812 15.334930 4.011186

Cobalt (II) tetrakis(4-methoxyphenyl) -porphyrin (CoPor) (H-absorped):

112 (no. of atoms)

C Co H N O H

C 3.133264 5.724166 14.757006

C 4.779472 7.336306 13.113931

C 4.928429 5.764449 2.569831

C 5.202834 6.854522 14.359716

C 5.683091 8.014430 12.291081

C 6.180474 7.122533 4.701054

C 6.525311 7.081446 14.772773

C 6.900479 6.329463 3.797009

C 6.858133 7.773272 5.735098

C 7.019148 8.223958 12.670361

C 7.413312 7.751077 13.938671

C 7.512268 7.107666 10.025001

C 7.758749 6.994164 8.690227

C 8.289241 6.191713 3.953406

C 7.988063 8.870143 11.746175

C 8.246574 7.660953 5.899835

C 8.153480 8.320743 10.466710

C 8.563985 8.126938 8.317590

C 8.947425 6.851261 4.987674

C 8.575497 10.598584 13.461920

C 8.726200 9.976829 12.175506

C 8.908079 8.424221 6.996840

C 9.631463 11.448821 13.613466

C 9.757933 9.485781 6.674441

C 10.154442 9.811913 5.328944

C 10.405203 11.386169 12.398416

C 11.039019 10.844918 5.420974

C 11.142606 11.182623 6.815566

C 11.585469 12.141714 12.185199

C 11.881812 12.537949 10.856968

C 11.918778 12.236410 7.300968

C 11.968971 12.587391 8.652288

C 12.401276 11.560870 14.483801

C 12.581784 9.133746 10.949470

C 12.457209 12.419599 13.358138

C 12.538896 8.588970 8.698173

C 12.292213 13.832189 5.358785

C 12.652856 13.696344 10.476937

C 12.695866 13.732064 9.119555

C 12.800430 12.996913 6.367415

C 13.175354 11.747423 15.631987

C 13.147761 14.585247 4.551152

C 13.451397 13.429121 13.439939

C 13.897576 8.793768 11.076590

C 13.847638 8.202357 8.707209

C 14.098314 12.797992 15.688183

C 14.246354 13.615119 14.558939

C 14.195228 12.918996 6.528569

C 14.716560 8.337218 9.915100

C 14.531358 14.511187 4.744134

C 15.056506 13.666523 5.726084

C 14.693408 12.376536 17.967968

C 15.536497 7.042532 10.200300

C 15.701319 14.826638 2.710541

C 16.596105 7.210520 11.286855

Co 10.182160 10.343063 9.562470

H 2.697787 5.130527 15.568190

H 2.469343 6.574898 14.531617

H 3.755011 7.189326 12.774746

H 4.620480 6.809329 2.399775

H 3.239462 5.097079 13.857003

H 4.688282 5.164939 1.684868

H 5.103139 7.249222 4.606378

H 4.387638 5.362067 3.442343

H 5.339769 8.385807 11.324718

H 6.845197 6.697941 15.742246

H 6.292556 8.399175 6.427056

H 6.952121 6.432579 10.662154

H 7.439204 6.212533 8.008931

H 8.447421 7.885307 14.258233

H 7.762316 10.409485 14.153792

H 8.834576 5.557174 3.253536

H 10.025899 6.727969 5.097990

H 9.842348 12.111090 14.445685

H 9.806458 9.303122 4.436537

H 11.211913 13.915486 5.230542

H 11.739097 10.697409 14.463327

H 11.570948 11.346228 4.619222

H 12.018254 9.466467 11.820441

H 11.950175 8.506814 7.784128

H 13.062307 11.051733 16.463206

H 12.744519 15.260311 3.794515

H 13.011820 14.479207 11.128112

H 13.149408 14.488975 8.488994

H 13.637950 14.098692 12.613155

H 14.363726 8.872416 12.059615

H 14.284760 7.791342 7.794717

H 13.662971 12.511257 18.335565

H 14.602170 12.270727 7.305428

H 14.996052 14.407202 14.576862

H 15.497288 9.110277 9.706442

H 14.791554 14.713506 2.096062

H 14.834753 6.237910 10.467848

H 15.398748 12.802274 18.690503

H 16.137609 13.620404 5.866294

H 14.902012 11.301103 17.846816

H 16.016924 6.740441 9.255652

H 16.153185 7.464716 12.260580

H 16.365418 15.565392 2.246773

H 16.217510 13.852746 2.769211

H 17.311777 8.004935 11.025405

H 17.164955 6.280640 11.423550

H 9.124925 11.321381 9.367033

N 8.867689 8.890867 9.430924

N 9.802423 10.534451 11.494524

N 10.368944 10.333184 7.581099

N 11.409357 11.906856 9.717053

N 11.870148 9.103643 9.780062

O 4.408967 6.156551 15.232359

O 6.341648 5.657308 2.741067

O 14.905078 13.093636 16.752665

O 15.393859 15.319364 4.017693

Cobalt(II) phthalocyanine (CoPht)

81 (no. of atoms)

C Co H N

C 10.379285 10.432479 12.274381

C 10.415741 8.662961 6.686212

C 11.016376 11.046435 14.723843

C 10.152215 12.074310 8.325120

C 11.236503 10.550207 13.432789

C 12.050651 10.930196 15.650746

C 10.837255 13.182875 7.456129

C 12.322472 9.486288 11.718277

C 12.464288 9.949005 13.080963

C 12.239170 7.686884 7.958972

C 12.029978 12.608737 6.683099

C 13.281339 10.332763 15.296986

C 12.352496 11.549212 8.995378

C 13.110782 8.265519 9.910421

C 13.503232 9.835207 14.015065

C 12.639824 13.584843 5.667515

C 13.524613 7.028072 8.019484

C 13.055192 12.088047 7.713822

C 13.652565 12.927005 4.723199

C 14.088963 7.422841 9.254941

C 14.236887 6.227648 7.117999

C 15.385412 7.031524 9.603069

C 15.527407 5.835959 7.473323

C 16.094676 6.239960 8.699617

C 9.695528 7.979600 4.239696

C 9.468787 8.339431 5.563212

C 8.285982 8.879850 7.471811

C 8.579622 7.773471 3.416026

C 8.283506 10.529528 11.307368

C 8.170651 8.495202 6.058938

C 7.407491 9.729697 9.408127

C 7.277904 7.943338 3.910234

C 7.055417 8.311681 5.240040

C 6.843776 10.719266 11.403284

C 6.293749 10.197126 10.220869

C 6.023471 11.223669 12.414720

C 4.915390 10.174458 10.001992

C 4.641943 11.200316 12.201183

C 4.093922 10.685391 11.009344

Co 10.349134 9.436461 9.497458

H 9.265305 11.679686 7.805476

H 10.705526 7.875926 3.836257

H 9.802428 12.537471 9.272654

H 10.945753 9.630806 6.535201

H 10.088229 13.612287 6.773028

H 11.908200 11.297915 16.668113

H 11.620995 7.009521 6.141551

H 11.658684 11.743079 6.105816

H 11.186169 14.005464 8.101426

H 11.827439 14.027541 5.067188

H 13.191977 12.087448 4.178080

H 12.222366 12.366179 9.735269

H 12.988745 10.791231 9.458819

H 14.067501 10.252199 16.049145

H 13.813690 5.921810 6.158310

H 13.117994 14.423987 6.202597

H 14.445519 9.358640 13.741983

H 13.739282 12.904490 8.008151

H 13.672954 11.291710 7.273450

H 14.027157 13.640515 3.976196

H 14.524213 12.532462 5.265188

H 15.823486 7.353183 10.548816

H 16.111404 5.220452 6.788315

H 17.112533 5.929537 8.939995

H 10.057037 11.490905 14.989134

H 8.726588 7.488099 2.374027

H 6.452693 11.610754 13.338609

H 6.428980 7.790070 3.242822

H 6.050354 8.451435 5.638014

H 4.504563 9.761758 9.079542

H 3.972182 11.583902 12.971944

H 3.010705 10.676954 10.880133

N 11.054791 9.793490 11.243750

N 11.377285 7.608936 6.920539

N 11.091236 10.977257 8.544452

N 11.990057 8.413147 9.065156

N 13.270316 8.779067 11.102982

N 9.569162 8.873454 7.864717

N 9.102209 10.803726 12.295660

N 8.600398 9.930868 10.085896

N 7.220945 9.228866 8.195675

Cobalt(II) phthalocyanine (CoPht) (H-absorped)

82 (no. of atoms)

C Co H N H

C 10.385133 10.366561 12.291132

C 10.437384 8.657812 6.669443

C 11.040295 11.053848 14.719809

C 10.121704 12.139617 8.325981

C 11.248369 10.505116 13.449881

C 12.086809 10.975472 15.639429

C 10.819215 13.223900 7.472628

C 12.318225 9.399216 11.753131

C 12.471408 9.890045 13.109281

C 12.254466 7.673566 7.951259

C 11.985441 12.614540 6.683658

C 13.312957 10.366027 15.297647

C 12.350368 11.604099 9.020101

C 13.121049 8.243710 9.906447

C 13.521976 9.819082 14.031011

C 12.597173 13.590007 5.667695

C 13.541211 7.012851 8.011332

C 13.019165 12.073860 7.691110

C 13.646185 12.958564 4.747642

C 14.104216 7.409712 9.246991

C 14.256324 6.207300 7.117617

C 15.393949 7.010775 9.606615

C 15.542967 5.808939 7.483988

C 16.105263 6.209394 8.712727

C 9.703453 7.986646 4.220581

C 9.483079 8.335693 5.547923

C 8.303619 8.867886 7.469915

C 8.583381 7.783715 3.400184

C 8.292062 10.503197 11.307625

C 8.187016 8.486711 6.052620

C 7.412376 9.706257 9.412471

C 7.284387 7.937399 3.905387

C 7.068672 8.296095 5.240465

C 6.850188 10.711232 11.405123

C 6.294459 10.187852 10.224451

C 6.042519 11.243857 12.411432

C 4.915328 10.183756 10.012258

C 4.659434 11.242292 12.201121

C 4.103176 10.719057 11.017957

Co 10.343651 9.321746 9.529573

H 9.322926 11.648591 7.741702

H 10.710905 7.884063 3.810694

H 9.592762 12.633543 9.173700

H 10.985985 9.609025 6.479630

H 10.080791 13.682615 6.796649

H 11.956162 11.385391 16.642494

H 11.639633 7.001797 6.132734

H 11.577334 11.756456 6.114839

H 11.201984 14.038056 8.111734

H 11.786366 14.011685 5.051015

H 13.221840 12.115608 4.179301

H 12.347077 12.465295 9.725454

H 12.968075 10.827575 9.485593

H 14.108179 10.319840 16.043364

H 13.839983 5.897141 6.156989

H 13.045226 14.442079 6.210512

H 14.466013 9.343390 13.762074

H 13.750539 12.864245 7.935268

H 13.588432 11.240509 7.253848

H 14.026812 13.688594 4.018233

H 14.510270 12.576771 5.310270

H 15.824326 7.331744 10.555555

H 16.125953 5.182820 6.807289

H 17.117970 5.891182 8.963078

H 10.087892 11.522095 14.974895

H 8.724702 7.506226 2.354798

H 6.482214 11.638911 13.328739

H 6.431214 7.783861 3.242504

H 6.063941 8.424543 5.644925

H 4.491243 9.770080 9.096460

H 3.996944 11.646823 12.968820

H 3.019155 10.726845 10.890576

H 9.899132 8.028603 10.058508

N 11.052202 9.707681 11.286259

N 11.386921 7.594713 6.914619

N 11.011769 11.117324 8.823308

N 12.012061 8.403322 9.055535

N 13.268303 8.714167 11.123257

N 9.588416 8.892744 7.841973

N 9.109293 10.760217 12.304446

N 8.593566 9.924796 10.081723

N 7.230972 9.186211 8.203662

Vitamin B_12_ (CoVB_12_)

159 (no. of atoms)

C Co H N O

C 11.232208 12.775902 12.047890

C 11.864617 13.735195 14.388038

C 11.266384 9.295173 12.459654

C 11.419601 16.598140 9.051920

C 13.781149 12.478516 4.009277

C 12.292920 12.895201 13.169039

C 11.794885 7.823251 12.604250

C 14.828023 11.790388 6.183378

C 12.323766 11.391637 13.602948

C 12.083223 10.603137 12.283454

C 12.376325 7.546766 14.000256

C 14.268188 12.801181 5.398356

C 15.287423 12.076963 7.493599

C 12.608376 6.077095 14.382783

C 13.329154 10.935055 14.686234

C 12.813425 7.378024 11.537571

C 13.674748 13.449302 12.646893

C 16.211162 12.093463 9.518011

C 13.622109 14.240780 11.330169

C 14.155768 14.134061 5.890993

C 13.282068 10.461596 11.338694

C 15.086247 13.414037 7.985865

C 13.603727 15.233837 5.022759

C 14.555635 14.430935 7.212620

C 14.836953 11.183101 14.607340

C 14.908906 14.994688 11.046975

C 10.440723 7.082039 12.296119

C 10.503185 13.857598 11.431904

C 9.923562 16.269687 9.202090

C 9.903044 7.962607 11.166538

C 9.960716 13.705378 10.165169

C 10.257806 15.131679 12.205023

C 10.320941 14.324354 6.875792

C 9.477158 7.042204 13.511966

C 9.375637 14.812937 9.277102

C 9.213199 12.654581 8.321754

C 9.098873 7.522854 10.068489

C 9.185263 14.107703 7.894007

C 9.165921 7.181119 6.845310

C 8.712026 11.638881 7.521489

C 8.763567 8.387867 9.032055

C 8.565032 4.652993 13.471522

C 8.604388 10.298114 7.864132

C 8.704377 6.063210 10.014364

C 8.023855 10.957232 12.868435

C 8.229336 6.140257 13.397310

C 8.125636 7.957024 7.687799

C 7.866778 9.317497 7.008840

C 7.119865 11.861898 10.913792

C 7.851152 14.527541 7.257404

C 6.740761 10.182146 13.316705

C 6.815346 7.127173 7.764660

C 5.709808 11.603063 11.549995

C 5.680753 10.214250 12.200578

C 5.717923 7.687521 8.669832

C 4.288737 9.731066 12.636333

C 3.559751 10.568287 13.689290

Co 9.910503 10.770105 10.429938

H 11.304039 14.054872 7.279208

H 11.162562 15.716236 12.430433

H 10.654435 6.053292 11.985795

H 11.356983 11.267911 14.127027

H 11.916560 14.811510 14.193369

H 14.926924 10.775921 5.793276

H 11.755179 8.003970 14.788319

H 12.560037 13.537964 15.216408

H 13.872328 11.404796 3.800790

H 11.526604 17.227629 11.041726

H 14.349828 13.022114 3.237900

H 13.039103 11.454387 15.611725

H 13.148148 9.868463 14.878347

H 13.370548 13.585814 10.486498

H 12.478648 7.630163 10.523936

H 15.935826 11.632637 10.484746

H 12.853662 15.019597 11.384679

H 12.575448 15.018378 4.685921

H 12.931569 6.289032 11.598748

H 13.430012 6.693820 16.212159

H 13.375803 4.973475 15.935107

H 13.358552 8.041725 14.073417

H 12.994903 17.367712 10.120145

H 13.000343 9.899518 10.440631

H 14.105152 14.086442 13.431733

H 14.124547 9.948033 11.818016

H 13.800530 7.832996 11.686553

H 14.386802 12.624789 12.523265

H 17.308069 12.308418 9.566023

H 13.634285 11.440363 11.001652

H 14.204158 15.372694 4.108123

H 15.209727 9.260679 13.851656

H 13.577093 16.187013 5.565352

H 14.404690 15.435137 7.611285

H 16.558359 15.064589 9.834787

H 16.610466 10.251909 14.155444

H 12.724242 12.760949 3.873817

H 10.647678 9.474759 13.361956

H 10.846149 13.487133 14.722704

H 10.036442 6.705882 14.398011

H 9.534348 16.843774 10.053104

H 10.058736 7.801914 6.685736

H 10.379608 15.370681 6.551495

H 9.446857 16.716776 8.314682

H 10.129414 13.694712 5.994496

H 9.793738 14.897760 13.172804

H 8.910259 10.483620 13.316009

H 9.548975 15.777961 11.680399

H 9.490862 6.243547 7.314444

H 9.119779 8.059768 13.731396

H 9.237375 5.453487 10.749172

H 8.896348 5.611662 9.034277

H 8.741012 6.936030 5.859659

H 8.344575 14.946812 9.659897

H 8.560066 3.080427 14.791309

H 8.296549 11.925621 6.555978

H 7.981497 11.997560 13.279432

H 8.208237 9.335454 5.963163

H 6.993440 9.132128 13.542614

H 7.537637 6.410307 14.210517

H 7.700409 6.328002 12.454278

H 7.398710 12.924428 11.127783

H 7.898114 4.552701 15.454386

H 7.634660 5.926340 10.231501

H 7.836568 15.615414 7.088534

H 7.053686 11.752517 9.822442

H 7.703841 14.052129 6.277675

H 6.351707 10.629134 14.248060

H 6.995760 14.265294 7.896047

H 7.031262 6.083359 8.021696

H 6.796098 9.573833 7.026073

H 6.018728 9.512637 11.422101

H 6.390105 7.111495 6.746547

H 5.489643 12.376804 12.303708

H 4.936350 11.682667 10.770515

H 5.384536 5.803800 9.521299

H 4.119822 10.620220 14.634322

H 4.387071 8.695389 13.008294

H 3.390368 11.598368 13.345389

H 4.352186 7.096158 10.079697

H 3.660155 9.670434 11.731735

H 2.575939 10.133954 13.917634

N 10.349245 9.202373 11.301441

N 11.094054 11.530882 11.627823

N 15.883498 11.268109 8.389772

N 12.003335 17.152480 10.153645

N 13.175551 5.915007 15.618848

N 15.469719 13.372365 9.324167

N 15.738645 14.494178 10.066059

N 15.603244 10.135918 14.170173

N 9.749585 12.475358 9.551380

N 9.053250 9.742327 9.014462

N 8.092891 10.918553 11.426607

N 8.309525 4.055771 14.675899

N 5.118109 6.778718 9.495918

O 12.047658 16.466194 7.998108

O 12.320837 5.113344 13.673103

O 15.349731 12.234620 14.995658

O 15.212910 16.037091 11.639975

O 9.052199 4.020130 12.530125

O 5.356369 8.867453 8.646860

Vitamin B_12_ (CoVB_12_) (H-absorped)

160 (number of atoms)

C H C Co H N O

C 11.244723 12.761360 12.031533

C 11.831435 13.742975 14.378538

C 11.270432 9.288389 12.474380

C 11.446069 16.597260 8.980883

C 13.703978 12.482651 4.026183

C 12.283597 12.893609 13.176095

C 11.782722 7.809975 12.614073

C 14.775513 11.820246 6.197040

C 12.303793 11.391544 13.618795

C 12.103531 10.594002 12.295655

C 12.371171 7.531009 14.006023

C 14.219618 12.824568 5.402282

C 15.256089 12.120647 7.495752

C 12.609336 6.060352 14.381943

C 13.278002 10.930513 14.726897

C 12.782974 7.351958 11.535504

C 13.669492 13.454727 12.670342

C 16.187271 12.142304 9.516579

C 13.638203 14.224900 11.339945

C 14.138775 14.168043 5.873839

C 13.341869 10.424919 11.408622

C 15.079187 13.466969 7.972652

C 13.606663 15.261270 4.986144

C 14.557047 14.479401 7.186488

C 14.785521 11.168143 14.671247

C 14.921666 14.993671 11.080822

C 10.418307 7.080263 12.320313

C 10.514455 13.840376 11.402490

C 9.958677 16.261419 9.172361

C 9.872215 7.970205 11.199750

C 10.001441 13.693677 10.122916

C 10.238365 15.110939 12.170916

C 10.296783 14.311521 6.810998

C 9.471792 7.025585 13.546802

C 9.410021 14.803896 9.241118

C 9.237326 12.645587 8.289070

C 9.052919 7.532210 10.104275

C 9.189739 14.099629 7.862427

C 9.118444 7.214496 6.888819

C 8.709040 11.626682 7.509577

C 8.700815 8.397911 9.077023

C 8.557359 4.633734 13.483448

C 8.556607 10.298841 7.889587

C 8.658071 6.073512 10.055970

C 8.093005 11.019120 12.873083

C 8.223942 6.122816 13.435683

C 8.061593 7.966921 7.731597

C 7.781491 9.326090 7.062203

C 7.191007 11.925263 10.892060

C 7.838256 14.516305 7.259932

C 6.856854 10.136052 13.232657

C 6.763954 7.120608 7.793450

C 5.806993 11.631420 11.543946

C 5.802307 10.208809 12.111573

C 5.633723 7.682905 8.658077

C 4.411988 9.691455 12.522135

C 3.677646 10.497476 13.597261

Co 9.989381 10.753654 10.370700

H 11.289152 14.033619 7.185587

H 11.123363 15.736415 12.370369

H 10.626389 6.055384 11.994994

H 11.321568 11.274362 14.114233

H 11.880205 14.816699 14.170783

H 14.851663 10.797315 5.825143

H 11.750715 7.983445 14.797975

H 12.520213 13.559259 15.215584

H 13.720469 11.397524 3.858758

H 11.624897 17.204318 10.974576

H 14.301884 12.957361 3.230731

H 12.971581 11.455867 15.643897

H 13.080779 9.866530 14.914374

H 13.414782 13.555279 10.500088

H 12.444727 7.621114 10.527112

H 15.884483 11.698494 10.483335

H 12.858145 14.994290 11.364714

H 12.561795 15.076607 4.685733

H 12.873853 6.259773 11.586452

H 13.429971 6.669822 16.215599

H 13.383923 4.950428 15.926661

H 13.351939 8.029760 14.075621

H 13.055533 17.365048 10.000203

H 13.093673 9.865725 10.500206

H 14.075840 14.109930 13.452367

H 14.152386 9.902085 11.932213

H 13.782476 7.780842 11.680585

H 14.392929 12.636706 12.579249

H 17.288673 12.324055 9.577805

H 13.722043 11.397507 11.085629

H 14.187360 15.346158 4.052118

H 15.180137 9.203045 14.041905

H 13.631490 16.231345 5.497190

H 14.433688 15.494853 7.565262

H 16.573402 15.109943 9.869848

H 16.568804 10.216860 14.311581

H 12.667928 12.828536 3.883655

H 10.664668 9.473083 13.384140

H 10.810304 13.492420 14.702071

H 10.041990 6.675676 14.420307

H 9.592306 16.821043 10.043318

H 10.009679 7.842689 6.753959

H 10.349910 15.357921 6.485195

H 9.460317 16.721979 8.304446

H 10.073032 13.684974 5.934997

H 9.802455 14.870937 13.149480

H 8.995416 10.595688 13.340085

H 9.495496 15.724890 11.654873

H 9.437795 6.269361 7.345649

H 9.118361 8.040441 13.785580

H 9.196752 5.464717 10.786711

H 8.840739 5.616859 9.076732

H 8.708096 6.989158 5.892944

H 8.387212 14.941025 9.644512

H 8.528703 3.030771 14.765799

H 8.289242 11.904794 6.543375

H 7.949248 12.019234 13.362306

H 8.073884 9.347296 6.002247

H 7.165435 9.086543 13.371811

H 7.542791 6.381538 14.261134

H 7.682012 6.323657 12.503247

H 7.379813 13.029267 10.997504

H 7.865382 4.489940 15.455949

H 7.590065 5.934513 10.283039

H 7.824251 15.603719 7.088299

H 7.125922 11.732589 9.810234

H 7.665638 14.039009 6.285984

H 6.431984 10.477498 14.193317

H 7.000129 14.257212 7.921869

H 6.986110 6.085664 8.080238

H 6.712182 9.581498 7.130295

H 6.150630 9.553658 11.295961

H 6.364312 7.078484 6.765632

H 5.604073 12.353002 12.352206

H 5.008911 11.762484 10.794582

H 5.383403 5.841905 9.623363

H 4.244081 10.532104 14.539578

H 4.517665 8.645781 12.867197

H 3.506263 11.535053 13.276253

H 4.284894 7.115548 10.092015

H 3.785923 9.652836 11.613961

H 2.696290 10.053747 13.820537

H 11.168193 10.481712 9.519958

N 10.333872 9.196646 11.331621

N 11.145159 11.519929 11.613621

N 15.850632 11.315477 8.394097

N 12.074440 17.117424 10.073874

N 13.188169 5.894113 15.612597

N 15.487176 13.436979 9.300246

N 15.735863 14.550127 10.057312

N 15.562432 10.095817 14.322730

N 9.798223 12.473741 9.501795

N 8.989774 9.751048 9.043768

N 8.261875 11.139100 11.453061

N 8.284761 4.010206 14.670739

N 5.061708 6.795459 9.524658

O 12.029417 16.502404 7.897363

O 12.318649 5.098883 13.670160

O 15.289263 12.242825 15.002279

O 15.234388 16.002395 11.722137

O 9.053620 4.017846 12.535589

O 5.220249 8.843293 8.564738
